# Evidence for Human Streptococcus pneumoniae in wild and captive chimpanzees: A potential threat to wild populations

**DOI:** 10.1038/s41598-017-14769-z

**Published:** 2017-11-06

**Authors:** Sophie Köndgen, Sebastien Calvignac-Spencer, Kim Grützmacher, Verena Keil, Kerstin Mätz-Rensing, Kathrin Nowak, Sonja Metzger, John Kiyang, Antina Lübke-Becker, Tobias Deschner, Roman M. Wittig, Felix Lankester, Fabian H. Leendertz

**Affiliations:** 1 Epidemiology of highly pathogenic microorganisms, Robert Koch-Institute, 13353 Berlin, Germany; 2 Institute of Medical Virology, Charité – Universitätsmedizin Berlin, 10117 Berlin, Germany; 3 Department of Pathology, German Primate Center, 37077 Göttingen, Germany; 4 Max Planck Institute for Evolutionary Anthropology, Department of Primatology, 04103 Leipzig, Germany; 5 Limbe Wildlife Centre, Limbe, SW Region Cameroon; 6 Berlin Institute of Microbiology and Epizootics, Freie Universität Berlin, 14163 Berlin, Germany; 7 Taï Chimpanzee Project, Centre Suisse de Recherches Scientifiques, 01 BP 1303 Abidjan, Ivory Coast; 8 Paul G. Allen School for Global Animal Health, Washington State University, Pullman, WA 99164 USA; 9 Present Address: Department for Infectious Disease Epidemiology, Robert-Koch-Institute, 13353 Berlin, Germany; 10 Present Address: Evolutionary Ecology, Leipniz Institute for Zoo and Wildlife Research, 10315 Berlin, Germany

**Keywords:** Zoology, Conservation biology, Bacterial infection

## Abstract

Habituation of wild great apes for tourism and research has had a significant positive effect on the conservation of these species. However, risks associated with such activities have been identified, specifically the transmission of human respiratory viruses to wild great apes, causing high morbidity and, occasionally, mortality. Here, we investigate the source of bacterial-viral co-infections in wild and captive chimpanzee communities in the course of several respiratory disease outbreaks. Molecular analyses showed that human respiratory syncytial viruses (HRSV) and human metapneumoviruses (HMPV) were involved in the etiology of the disease. In addition our analysis provide evidence for coinfection with *Streptococcus (S.) pneumoniae*. Characterisation of isolates from wild chimpanzees point towards a human origin of these bacteria. Transmission of these bacteria is of concern because – in contrast to HRSV and HMPV - *S. pneumoniae* can become part of the nasopharyngeal flora, contributing to the severity of respiratory disease progression. Furthermore these bacteria have the potential to spread to other individuals in the community and ultimately into the population. Targeted vaccination programs could be used to vaccinate habituated great apes but also human populations around great ape habitats, bringing health benefits to both humans and wild great apes.

## Introduction

Due to their close genetic relationship, humans and other great apes share susceptibility to a considerable number of pathogens^1,2^ with the transmission of human respiratory pathogens to captive apes being a well-documented concern^3–11^. In this context, it had been long suspected that pathogen transmission from humans might account for disease outbreaks observed in wild great apes habituated to human presence for research and tourism^7,12–14^. This suspicion was confirmed when common human respiratory paramyxoviruses (human respiratory syncytial virus (HRSV) and human metapneumovirus (HMPV)) were identified in chimpanzee *(Pan troglodytes verus)* lung tissue and faeces collected during separate disease outbreaks in Taï National Park (Côte d’Ivoire)^15,16^. Further studies on wild chimpanzees in Tanzania *(Pan troglodytes schweinfurthii)* and gorillas *(Gorilla beringei)* in Rwanda and the Central African Republic have demonstrated additional incidences in which human respiratory paramyxoviruses have been transmitted to great apes indicating that these events are neither isolated nor a feature of a particular region (Table 1)^17–19^.

**Table 1:** Characteristics and analyses of respiratory disease outbreaks among chimpanzees in Taï National Park (Côte d’Ivoire) and Limbe Wildlife Center (Cameroon).

Location	Group	Year	Morbidity	Mortality	Samples collected	HRSV	HMPV	*S. pneumoniae* (Screening PCR)	*S. pneumoniae* (Culture)	*S. pneumoniae* (MLST)
Taï National Park	South Group	2009	86%	6*/37	Lung tissue (n = 6)	Type A	negative	Positive	accomplished (n = 2)	accomplished (n = 2; ST 8485)
Limbe Wildlife Center	Group 1	2006	100%	None	Pharyngeal swab (n = 2)	negative	Type A2	Positive	not tested	not tested
		2007	23%	None	n.s.	n.s.	n.s.	n.s.	n.s.	n.s.
	Group 2	2006	29%	None	Pharyngeal swab (n = 3)	negative	Type A2	Positive	not tested	not tested
		2007	100%	None	Pharyngeal swab (n = 1)	Type B	negative	Positive	not tested	not tested
	Group 3	2007	100%	1/4	Lung tissue (n = 1)	Type B	negative	Positive	not tested	accomplished (n = 1; ST 8949)

*Refers to the number of individuals where necropsies had been performed. Further five animals were found dead but necropsies were not performed due to the destruction of the carcass by scavengers or logistical reasons. Additionally three animals disappeared during the outbreak and are considered victims of the epidemic.

n.s. not sampled.

In addition to human viruses, the bacterium *Streptococcus pneumoniae* (or pneumococcus) was also detected during outbreaks where samples of lung tissue had been collected^15,18,20^, leading to the conclusion that it was the combination of respiratory viruses *and* pneumococci, and not the viral infection alone, that caused the fatalities^15,16^. Given that invasive pneumococcal infections are also a primary cause of severe pneumonia in humans worldwide^21,22^, these bacterial co-infections are a cause for concern since they could lead to increased mortality and persist in the great ape populations.

However the provenance of the pneumococci strains from the great ape cases mentioned above remains uncertain. Sequence analyses have so far only been performed on *S. pneumoniae* positive chimpanzee samples from Taï National Park and these showed differences to known human sequences^20,23^. Unfortunately, there is a lack of data on regional human pneumococci sequence types and therefore transmission from humans could not be confirmed^20^.

In this article we investigate respiratory disease outbreaks causing mortalities among wild and captive wild-borne great apes in West and western-Central Africa. Again, HRSV and HMPV were detected together with pneumococci and we provide evidence that all were of human origin. These results are important since, in contrast to human respiratory viruses, pneumococci may become an established part of the great ape oropharyngeal flora and could severely impact the outcome of subsequent respiratory infections or, as in humans, become the primary cause of severe pneumonia.

## Results and Discussion

Samples were collected in the course of several respiratory disease outbreaks that occurred at two different locations: in the Limbe Wildlife Center (LWC) situated in Cameroon, and in Taï National Park (TNP), Côte d’Ivoire.

The LWC is a wildlife rehabilitation project in which rescued great apes are kept in naturalistic enclosures. Here, a total of five distinct outbreaks of respiratory disease occurred over a period of two years (2006/2007), in three separate chimpanzee groups (see Table 1): group 1 consisted of 22 individuals (age >5years), group 2 of 14 individuals between two and five years of age and group 3 consisted of four individuals of less than two years of age. The chimpanzees showed varying clinical symptoms (coughing, serous to mucopurulent nasal discharge, lethargy, inappetence) over the course of the outbreaks. Symptoms indicative of lower respiratory tract infection, such as dyspnoea and wheezing sounds, were noted on auscultation of the lungs of 12 individuals. Duration of the outbreaks ranged from several days up to nine weeks. Morbidity ranged from 23 to 100% (with 100% in three of the five outbreaks; see Table 1). During these outbreaks pharyngeal swabs from six chimpanzees were taken under anesthesia. During one outbreak an infant chimpanzee (referred to as LWC infant) died, a necropsy was performed and tissue samples were taken. Throughout the outbreaks, animals with severe symptoms were placed on antiobiotic therapy (see Supplementary Information).

In TNP, wild chimpanzees are habituated to the presence of human observers and individually known as a result of a project focusing on wild chimpanzee behaviour^24^. Here, three chimpanzee communities (so called North, East and South community) are under observation. In November 2009 the South community was affected by a respiratory disease outbreak. Morbidity and mortality were high: 32/37 chimpanzees developed severe respiratory symptoms (including high frequency of heavy cough, lethargy, nasal discharge). Four adults (>15 years; hereinafter referred to as TNP adult 1–4) and two infants (0–5 years, referred to as TNP infant 1 and 2) were found shortly after death and necropsies were performed. In addition, for one chimpanzee (adult) only one arm and the head was found due to scavenging. Four infants were found dead but necropsies were not performed and three further adults disappeared in the course of the outbreak and are likely victims of the outbreak as well. In total likely 8 adults and 6 infant chimpanzees died in this outbreak. Twelve out of the 37 individuals who showed severe symptoms of respiratory disease were treated with Extencilline (Benzathine benzylpenicillin, Sanofi-Aventis France), a long acting antibiotic administered by remote injection. Of these nine survived.

### Histopathology

**Figure 1:**
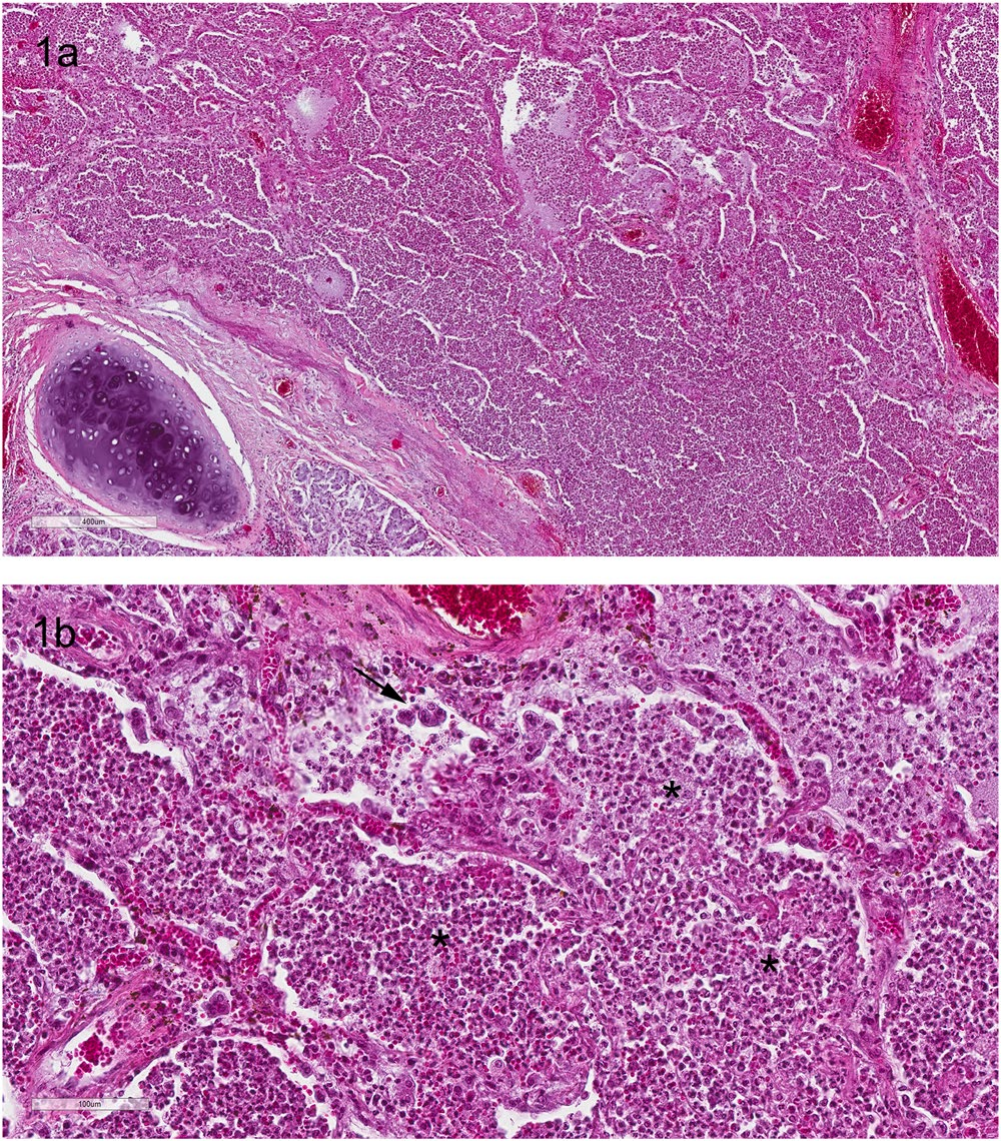
(**a**,**b**) Histopathology from lung of a chimpanzee from TNP, purulent bronchopneumonia. (**1a**) overview showed parts of a bronchus in the lower left and lung alveoli completely filled with exudate. Scale bare 400 µm. (**1b**) The exudate in the alveoli (asterisk) is composed of neutrophilic granulocytes, histiocytes and fibrin, the arrow points on cell syncytia, scale bar 100 µm. Hematoxylin and Eosin stain.

Histopathological examination of lung tissue samples from TNP and LWC chimpanzees revealed a severe purulent bronchopneumonia as cause of disease. The bronchoalveolar tissue was diffusely infiltrated with neutrophilic granulocytes and macrophages. Furthermore, all lung tissue samples collected during the TNP outbreak showed multinucleated cell syncytia, pointing towards an HRSV infection (Fig. 1). Additionally, gram-positive cocci could be demonstrated within the altered tissue giving evidence for a secondary bacterial infection. In addition, the lung tissue of one animal (TNP adult 3) revealed signs of a lung mite infection (likely *Pneumonyssus simicola*). The lung tissue from the LWC infant showed a multifocal to diffuse purulent bronchopneumonia with marginal alveolar histiocytosis. The periodic acid schiff (PAS) reaction was negative for all samples excluding a fungal infection.

### Molecular analysis

All samples collected from the TNP and LWC outbreaks were analysed molecularly by screening PCR and tested positive for either HMPV- or HRSV-specific genes as well as for the *lytA* gene harboured by *S. pneumoniae* (see Table 1). Phylogenetic analyses of generated paramyxovirus sequences revealed that both HRSV and HMPV sequences (Genbank accession no. JX489496- JX489498) belonged to recent pandemic waves (see Supplementary Figs 1–3). Informing a relaxed molecular clock model with tip dates^25^ we estimated that the LWC chimpanzee-infecting HMPV diverged from its closest human-infecting relatives in 2004 (95% highest posterior density: 2003–2004). The date of divergence between the HRSV-A strain from TNP chimpanzees and humans was 2007 (95% highest posterior density: 2006–2008) and for HRSV-B strains infecting LWC chimpanzees and humans 2007 (95% highest posterior density: 2007) These results mirror findings from previous outbreaks^15–19^ and emphasize the high incidence of anthropozoonotic transmission of human respiratory paramyxoviruses.

In order to gain new insights on the bacterial community present in the lung samples collected during the outbreak in TNP, we performed 16S rRNA PCR. Characterization of the sequences using a BLAST search in GenBank indicated the presence of *Streptococcus* species in all samples analysed. In addition, for one individual (TNP adult 3) *Pasteurella (P.) multocida* was also among the predominant BLAST hits. *P. multocida* is a commensal in the upper respiratory tracts of many domestic and wild animal species worldwide and coinfection with *P. multocida* and *S. pneumoniae* among TNP chimpanzees has been reported previously^16,20,26^. In addition, for the samples from TNP adult 1, the BLAST search revealed *Clostridium sp*. Since most species of the genus *Clostridium* are saprophytic, we conclude that this finding was due to the tissue samples being partly decayed.

Molecular analysis of the *S. pneumoniae* is considered separately in the next section.

### Phenotypic and molecular characterisation of *S. pneumoniae*

Isolation of pneumococci was achieved for TNP adult 1 and 2 (isolate IMT 113 and 115) from whom lung tissue samples had been stored adequately in Skim Milk-Tryptone-Glucose-Glycerol (STGG). Antibiotic susceptibility testing (Table 2) showed low resistance: isolates were resistant against sulfamethoxazole/trimethoprim (TMP-SMX) only. Further characterisation by Multi Locus Sequence Typing (MLST, see Table 3) indicated that alleles from isolates termed IMT 113 and 115 were identical and matched known alleles from human strains. Their allelic profile revealed 6 matches to various published serotype 19 A strains from Kenya and the Gambia (>100 isolates) but harbour a variant *ddl* allele. Therefore a new sequence type (ST 8485) was assigned (see Table 2). Serotyping of the TNP samples by PCR also revealed capsular type 19 A.

**Table 2:** Resistance pattern of pneumococcus isolates present in lung tissue of deceased TNP chimpanzees (adults 1 and 2).

Drug tested	IMT 113	IMT 115
chloramphenicol	S	S
enrofloxacin	S	S
oxacillin	S	S
sulfamethoxazole/trimethoprime	R	R
clarithromycin	S	S
erythromycin	S	S
vancomycin	S	S
clindamycin	S	S
tetracycline	S	S

**Table 3:** Allelic profile of *S. pneumoniae* originating from chimpanzee lung tissue compared to closely related human isolates from the MLST data base.

Isolate/DNA sample (country of origin)	Allel number for gene fragment							ST	Serotype
	*aroE*	*gdh*	*gki*	*recP*	*spi*	*xpt*	*ddl*		
**IMT113/115 (TNP, Côte d’Ivoire)**	7	11	4	1	6	112	8	8485*	19 A
107 isolates (Kenia and Gambia)	7	11	4	1	6	112	14	847	19 A
PVT0032 (Gambia)	7	11	4	1	6	112	15	3326	19 A
**LWC infant (LWC, Cameroon)**	177	134	4	18	9	83	18	8949	n.d.
KSA08 (Chad)	177	134	4	18	9	83	18	8949	9 V

Samples analysed in this study are written in bold.

n.d. not detected.

*New ST.

Lung samples from the LWC infant had not been stored adequately for cultivation. Therefore, a full DNA extract of this sample was used for further characterization. Hence, MLST results are based on “virtual clones” (see methods). For the LWC sample all seven alleles matched known alleles from human strains and indeed, allelic profiles were identical to a serotype 9 V strain isolated from a human of African origin (see Table 2). DNA from the LWC sample was tested for capsular type 9 V^27^ but tested negative. In addition we tried another 9 V/9 A primer combination described by Carvalho *et al*.^28^ but again the DNA tested negative. Unfortunately due to lack of DNA material it was not possible to test for further serotypes.

In summary, the MLST profiles of the strains described here showed high similarities or were even identical to human isolates, especially in Africa. In addition, results from the antibiotic susceptibility testing (Table 2) showed that the isolates from TNP chimpanzees were resistant to TMP-SMX. Resistance to TMP-SMX, which has been used widely in developing countries, is observed frequently and globally in human strains of *S. pneumoniae*^29^. The fact that this drug has never been used in the chimpanzees of TNP supports the assumption that the *S. pneumoniae* strain found originated in humans.

*S. pneumoniae* infection, especially in combination with respiratory viruses, can lead to invasive lower respiratory disease with fatal outcomes. Furthermore, and in contrast to respiratory virus infections, which are usually temporary, *S. pneumonia* infection in humans can result in an asymptomatic colonization of the nasopharynx. Additionally this bacterium can spread horizontally and remain silent within a population^30^. In fact, pneumococcal disease in humans will not occur without preceding nasopharyngeal colonization^30^, hence an increased rate of colonization within a population will increase the incidence of invasive pneumococcal disease. To investigate whether the TNP chimpanzee population are ‘carriers’ of *S. pneumoniae*, we investigated a throat swab obtained in 2011 during a necropsy on an adult female chimpanzee living in TNP (named “XENA”; East group). This chimpanzee had died from an anthrax-like disease^31^ independent of any respiratory disease outbreak. Since no isolate of *S. pneumoniae* could be obtained, we tested full DNA for the presence of the new variant of the capsule 3 (cps3Taï) described in chimpanzees that died between 2004 and 2006^23^ and performed MLST analyses. Indeed, the sample was positive for cps3Taï. MLST revealed several sequences with close matches to *S. pneumoniae* but also *S. oralis* and *S. mitis*. The finding of mixed sequences of oral commensal bacteria was not unexpected since we used a throat swab (instead of a pure culture of *S. pneumoniae*) for examination. However, exact matches to all sequences characterizing the ‘South clone’ ST 2309 (found in the lung tissue of deceased TNP chimpanzees which died in respiratory disease outbreaks in TNP in 2004 and 2006), have been amongst the sequences obtained. This provides preliminary evidence that this ST may have circulated within the chimpanzee community for at least five years.

## Conclusion

Here we provide the first evidence that *S. pneumoniae* can be transmitted and that the origin of this pathogen might be human. *S. pneumoniae* has the potential to circulate over long periods of time in a given population (a finding supported by our data on the chimpanzee XENA) and may cause severe disease once an individual is weakened by further primary viral infections or through other circumstances^30,32^. Given that adolescent female chimpanzees migrate to other communities and that other inter-troop encounters occur, the reported findings could have serious implications, not only in respect to the clinical outcome of individuals infected, but also for populations of wild chimpanzees^33^. In addition, the risk of ex-captive chimps transmitting human *S. pneumoniae* into wild populations is a serious concern for reintroduction programs.

These results underline the importance of hygiene measures, as established in TNP and described in the new IUCN guidelines, in limiting the risk of pathogen transmission between humans and great apes^34^. Moreover these results underscore the importance of developing non-invasive sample collection methods to assess the risk of the spread of such pathogens to wild chimpanzee and other great ape communities. We note that, characterisation of *S. pneumoniae* present in human communities in contact to or neighbouring wild great ape populations are needed to allow further conclusions regarding naturally occurring, and introduced, infections. Given the availability of vaccines that provide protection against the serotypes most frequently associated with pneumococcal disease in humans, characterisation of the distribution of serotypes would be of great interest. Such data will inform the design of vaccination campaigns targeting human-habituated great apes but also human populations in contact with wild great apes, providing enormous benefits to both human and wild animal health.

## Material and Methods

### Sampling

Necropsies on chimpanzees were conducted under high safety standards and precautions such as protection suits, gloves, and face masks were used to avoid contamination of samples with human pathogens. At LWC pharyngeal swabs were collected from six animals under sedation. All samples were preserved in RNAlater (LWC) or liquid nitrogen (TNP) and shipped to Germany for detailed analyses. No experiments on live animals were performed for this study, samples were collected from animals found dead or sedated for treatment necessary to save their lives, and therefore no animal ethics permission is required according to the national laws of Ivory Coast and Cameroon.

### Histology

Tissue samples from five of the six deceased chimpanzees from TNP on which necropsies had been performed, and from the deceased chimpanzee from LWC were submitted to the German Primate Centre (Göttingen, Germany) for histopathological examination. All samples were fixed in 4% formalin, embedded in paraffin, cut in 2–4 µm tissue sections and stained with hematoxylin and eosin for microscopic examination. Tissue was also stained with Giemsa and Gram stain and to visualize fungal infection furthermore PAS reaction was performed.

### DNA/RNA extraction and cDNA synthesis

Nucleic acids from lung tissue were extracted using the Nucleo Spin Tissue - and Nucleo Spin RNA II Kit (Macherey-Nagel, Düren, Germany), according to the manufacturer’s instructions. Swab samples stored in RNAlater were vortexed vigorously and RNA and DNA were extracted simultaneously from 280 µl of RNAlater using the QIAamp viral Mini Kit (Qiagen, Hilden, Germany). cDNA was synthesized by using the Superscript Kit (Invitrogen, Karlsruhe, Germany) and random hexamer primers (TIB Molbiol, Berlin, Germany).

### PCR screening for respiratory pathogens and phylogenetic analysis

DNA or cDNA respectively were screened for influenza A&B, HMPV, HRSV, Adeno-, Picornavirus and *S. pneumoniae* using PCR assays as described previously^35–40^. For phylogenetic analysis, the G gene of HMPV or HRSV was amplified using published PCR assays^17,41^ PCR products were purified using ExoSAP (USB Europe GmBH, Staufen, Germany) and sequenced subsequently using the ABI Big Dye Termination Kit (Applied Biosystems, Weiterstadt, Germany). Phylogenetic analyses of the paramyxovirus sequences were performed in a Bayesian framework. First, data sets were assembled that comprised one exemplary sequence per outbreak (-year) generated in this study (see supplementary information for details) and human HMPV or HRSV G gene sequences extracted from publicly availably full genomes with known isolation dates (retrieved from NCBI). Both data sets were aligned using MUSCLE^42^, as implemented in SeaView, version 4^43^, and reduced to only include unique sequences using FaBox^44^. Conserved alignment blocks were selected using gblocks (also implemented in SeaView). Three final alignments were generated: HMPV with 129 sequences and 711 positions, HRSV-A with 284 sequences and 968 positions and HRSV-B with 139 sequences and 954 positions. To determine the appropriate nucleotide substitution model, we estimated model likelihoods with jModeltest v2.1.10^45^ and compared them using the Bayesian information criterion (BIC). The selected models were GTR + G for HMPV, GTR + I + G for HRSV-A and HKY + G for HRSV-B. Bayesian analyses were run in BEAST v1.8.2^46^ assuming a longnormal-relaxed clock and a constant population size. Three chains were run for 100 million generations; convergence of the runs and appropriate sampling of the posterior were assessed using Tracer v1.6^47^. Post burn-in trees from the three chains were combined using LogCombiner v1.8.2 before being summarized onto the maximum clade credibility tree identified with TreeAnnotator v1.8.2 (both software programs are distributed with BEAST). Branch robustness was assessed through posterior probabilities.

### 16s rDNA Libraries from lung tissues

For a first screening for relevant bacteria, DNA from lung tissue samples from the TNP cases were tested using a 16s PCR^48^. Insufficient material was available for these analyses to be performed on the samples from the LWC infant. Amplicons were cloned into a pCR™ 2.1-TOPO® vector (Invitrogen, Karlsruhe, Germany) and colony PCR was performed on ten different clones (per sample) using M13 primers. PCR products were purified using ExoSAP (USB Europe GmBH, Staufen, Germany) and sequenced using the ABI Big Dye Termination Kit (Applied Biosystems, Weiterstadt, Germany). Sequences were compared to sequences contained in Genbank using the BLAST algorithm (http://blast.ncbi.nlm.nih.gov/Blast.cgi).

### Characterization of *S. pneumoniae*

#### Isolation

Lung tissue preserved in STGG had been collected from TNP adult 1 and 2 and was streaked on tryptic soy yeast extract (TSYE) agar supplemented with 5% of defibrinated sheep blood (Oxoid, Wesel, Germany) and incubated overnight in 5% CO_2_. *S. pneumoniae* was identified by colony morphology, α-hemolysis and optochin sensitivity and confirmed by PCR analysis as described below. Isolates from TNP adult 1 are registered under the number IMT 113 and the ones from TNP adult 2 as IMT 115.

#### Antimicrobial susceptibility testing

Antimicrobial susceptibility testing was performed using the agar diffusion test according to the standards given by the Clinical and Laboratory Standards Institute^49,50^. The antimicrobial agents tested included chloramphenicol (30 μg), clindamycin (2 μg), oxacillin (1 µg, sulfamethoxazole/trimethoprime (1, 25/23, 75 μg), tetracycline (30 μg), enrofloxacin (5 µg), erythromycin (15 µg), clarithromycin (15 µg) vancomycin (30 µg) (Becton Dickinson, Heidelberg, Germany).

#### Multi Locus Sequence Typing (MLST)

For MLST analysis, DNA was extracted from overnight cultures (TNP isolate IMT 113 and 115) or directly from lung tissue from the LWC sample using the Nucleo Spin Tissue Kit (Macherey & Nagel, Stadt, Land) according to the manufacturer’s instructions. PCR analyses were performed using standard primers and protocols given on the *Streptococcus pneumoniae* MLST website (http://pubmlst.org/spneumoniae) using a proof reading polymerase (Promega, Berlin, Germany). PCR fragments of the seven housekeeping genes *adh, gdh, gki, spi, recP, xpt and ddl* were obtained for all samples.

Amplicons from the TNP isolates were purified directly using ExoSAP (USB Europe GmBH, Staufen, Germany) and sequenced using the ABI Big Dye Termination Kit (Applied Biosystems, Weiterstadt, Germany). For the sample from LWC (DNA from lung tissue instead of a pure culture) MLST results were generated based on “virtual clones”: to minimize the possibility that two or multiple pneumococcus strains were involved, each of the seven amplicons was cloned into a pCR™ 2.1-TOPO® vector (Invitrogen, Karlsruhe, Germany). For every cloning reaction, ten clones were picked and colony PCR was performed using M13 primers. PCR products were purified and sequenced as described above. For every allele, sequences were checked for consistency using Genious version 7.1.4 (http://www.geneious.com)^51^.

Sequences obtained were entered in the MLST database and allelic numbers were assigned (all alleles were already present in the database). Since the TNP isolates showed a new allelic combination i.e. differed in one locus (*ddl*), a new ST was assigned by the curator (Table 2).

#### Serotyping PCR

Serotyping was done by PCR according to Pai *et al*.^27^. However, due to a shortage of DNA material, we did not follow the sequential multiplex PCR assay described, but rather chose serotype specific primers according to the serotype information given for human strains in the MLST database that matched our clones the most. DNA from TNP bacterial isolates were tested using the primers specific for capsule type 19 A. DNA from the LWC lung sample was tested for serotype 9 V but tested negative. In addition a 9 V/9 A primer combination described by Carvalho *et al*.^28^ was tried however the DNA tested negative. Further serotype testing was not possible due to a lack of DNA.

### Data availability

The datasets generated and analysed during the current study are available from the corresponding author on request.

### Accessions code

Sequences data on the HRSV and HMPV strains are available in Genbank under the accession number JX489496 - JX489498.

## Electronic supplementary material

Supplementary Information
