# Author Correction: Evidence for Human Streptococcus pneumoniae in wild and captive chimpanzees: A potential threat to wild populations

**DOI:** 10.1038/s41598-020-66183-7

**Published:** 2020-05-29

**Authors:** Sophie Köndgen, Sebastien Calvignac-Spencer, Kim Grützmacher, Verena Keil, Kerstin Mätz-Rensing, Kathrin Nowak, Sonja Metzger, John Kiyang, Antina Lübke-Becker, Tobias Deschner, Roman M. Wittig, Felix Lankester, Fabian H. Leendertz

**Affiliations:** 1 Epidemiology of highly pathogenic microorganisms, Robert Koch-Institute, 13353 Berlin, Germany; 2 Institute of Medical Virology, Charité – Universitätsmedizin Berlin, 10117 Berlin, Germany; 3 Department of Pathology, German Primate Center, 37077 Göttingen, Germany; 4 Max Planck Institute for Evolutionary Anthropology, Department of Primatology, 04103 Leipzig, Germany; 5 Limbe Wildlife Centre, Limbe, SW Region, Cameroon; 6 Berlin Institute of Microbiology and Epizootics, Freie Universität Berlin, 14163 Berlin, Germany; 7 Taï Chimpanzee Project, Centre Suisse de Recherches Scientifiques, 01 BP 1303 Abidjan, Ivory Coast; 8 Paul G. Allen School for Global Animal Health, Washington State University, Pullman, WA 99164 USA; 9 Present Address: Department for Infectious Disease Epidemiology, Robert-Koch-Institute, 13353 Berlin, Germany; 10 Present Address: Evolutionary Ecology, Leipniz Institute for Zoo and Wildlife Research, 10315 Berlin, Germany

Correction to: *Scientific Reports* 10.1038/s41598-017-14769-z, published online 06 November 2017

The original version of this Article contained a typographical error in the spelling of the author Antina Lübke-Becker, which was incorrectly given as Antina Lübke Becker. This has now been corrected in the PDF and HTML versions of the Article, and in the accompanying Supplementary Information file.

