# Supplementary material for: Evidence for Human Streptococcus pneumoniae in wild and captive chimpanzees: A potential threat to wild populations
Source: Sci Rep. 2017 Nov 6;7:14581. doi: 10.1038/s41598-017-14769-z (PMC5674046; doi:10.1038/s41598-017-14769-z)
Supplement: Supplementary file 1 — Supplementary Information [file 41598_2017_14769_MOESM1_ESM.pdf]

Supplementary Information

Evidence for Human Streptococcus pneumoniae in wild and captive chimpanzees: A potential threat to wild populations

Sophie Köndgen[1,2], Sebastien Calvignac-Spencer[1], Kim Grützmacher[1], Verena Keil[1], Kerstin Mätz-Rensing[3], Kathrin Nowak[1#], Sonja Metzger[4§], John Kiyang[5], Antina Lübke-Becker[6], Tobias Deschner[4], Roman M. Wittig[4,7], Felix Lankester[5,8], Fabian H. Leendertz[1]*

Affiliations:

[1] Epidemiology of highly pathogenic microorganisms, Robert Koch-Institute, Berlin, Germany

[2] Institute of Medical Virology, Charité – Universitätsmedizin Berlin, Berlin, Germany

[3] Department of Pathology, German Primate Center, Göttingen, Germany

[4] Max Planck Institute for Evolutionary Anthropology, Department of Primatology, Leipzig, Germany

[5] Limbe Wildlife Centre, Limbe, SW Region, Cameroon [6]

[6] Berlin Institute of Microbiology and Epizootics, Freie Universität Berlin, Germany

[7] Taï Chimpanzee Project, Centre Suisse de Recherches Scientifiques, Abidjan, Côte d'Ivoire

[8] Paul G. Allen School for Global Animal Health, Washington State University, Pullman, WA 99164, USA

* Correspondance to LeendertzF@rki.de

[#] Current affiliation: * Department for Infectious Disease Epidemiology, Robert-Koch-Institute, Berlin, Germany

[§] Current affiliation: Evolutionary Ecology, Leipniz Institute for Zoo and Wildlife Research, Berlin, Germany

Medical treatment at LWC

The first chimpanzee to fall sick (group 1) was treated with 1500mg of trimethoprim-sulphadiazine (Trimacare®, Animalcare Ltd., Dunnington, UK) per day orally for 10 days, initially, but deteriorated and was subsequently placed on intravenous fluid therapy (IFT) under sedation and a daily dose of 1000mg ceftriaxone (Lupin Pharmaceuticals, Inc., Baltimore, Maryland, USA), was administered parenterally for five days; after which the animal recovered. Throughout the following outbreaks, animals with severe symptoms were treated with either 30mg/kg amoxicillin-clavunate (Noroclavo®, Norbrook Laboratories Ltd., Co.Down, UK) three times per day or 5mg/kg enrofloxacin (Baytril®, Bayer HealthCare LLC, Kansas 66201, USA) orally. Nine animals in total with severe depression or dyspnoea were sedated and taken to the veterinary facility for intensive care therapy (IFT and 50mg/kg ceftriaxone per day, parenteral) until recovery. The infant that did not recover had additionally received 5ml of 2% mucolytic carbocysteine (Genemark Cameroun, Douala, Cameroon) orally.

Generation of HMPV and HRSV sequences

This section gives an overview about all the sequences that we generated:

<u>Limbe Wildlife Center:</u>

HMPV-A2 (2006): for HMPV, a 937 bp fragment of the P-gene was amplified (Mackay et al., 2004) and sequenced. The obtained sequences were compared and showed > 99 % nucleotide identity between individuals of group 1 and 2. The quality of these sequences was relatively low and did not allow us to cover the entire fragment on both strands so we did not deposit them in Genbank but provide them here (see below). These samples were analysed before we started to sequence the G gene for phylogenetic analysis. When we later aimed at generating G-sequences, most nucleic acids extracts were exhausted so we were only able to generate one G gene sequence from one individual. This sequence was deposited in Genbank (JX489498) and included exemplary in our dataset for subsequent phylogenetic analysis.

HRSV-B (2007): Amplicons of the G gene were generated and sequenced as described in the main text. Since sequences from group 2 (n=1) and group 3 (n=1) were identical, one exemplary sequence (JX489496) was deposited in Genbank and included in our dataset for subsequent phylogenetic analysis.

Tai National Park:

HRSV A (2009): Screening of the samples from TNP has been performed by qPCR (Reiche & Schweiger, 2009). Amplifying and sequencing of a G gene fragment has been done for one sample showing high copy numbers (JX489497).

Alignment of P gene consensus sequences

```
                                      1         10        20        30        40        50        60
                                      |         |         |         |         |         |         |
 chimp 350_group1 consensus sequence  -AAAGATATTCTTTTCATGGGTAATGAAGCAGCAAAATTGGCAGAGGCTTTTCAGAAATC
chimp 351_group 1 consensus sequence  -AAAGATATTCTTTTCATGGGTAATGAAGCAGCAAAATTGGCAGAGGCTTTTCAGAAATC
chimp 352_group 2 consensus sequence  -AAAGATATTCTTTTCATGGGTAATGAAGCAGCAAAATTGGCAGAGGCTTTTCAGAAATC
chimp 353_group 2 consensus sequence  -AAAGATATTCTTTTCATGGGTAATGAAGCAGCAAAATTGGCAGAGGCTTTTCAGAAATC
chimp 354_group 2 consensus sequence  AAAAGATATTCTTTTCATGGGTAATGAAGCAGCAAAATTGGCAGAGGCTTTTCAGAAATC

 chimp 350_group1 consensus sequence  ATTAAGAAAACCTAGTCATAAGAGATCTCAATCTATTATAGGAGAAAAAGTGAACACTGT
chimp 351_group 1 consensus sequence  ATTAAGAAAACCTAGTCATAAGAGATCTCAATCTACTATAGGAGAAAAAGTGAACACTGT
chimp 352_group 2 consensus sequence  ATTAAGAAAACCTAGTCATAAGAGATCTCAATCTATTATAGGAGAAAAAGTGAACACTGT
chimp 353_group 2 consensus sequence  ATTAAGAAAACCTAGTCATAAGAGATCTCAATCTATTATAGGAGAAAAAGTGAACACTGT
chimp 354_group 2 consensus sequence  ATTAAGAAAACCTAGTCATAAGAGATCTCAATCTATTATAGGAGAAAAAGTGAACACTGT

 chimp 350_group1 consensus sequence  ATCTGAAACATTGGAATTACCTACTATCAGTAGACCTACCAAACCAACCATATTGTCAGA
chimp 351_group 1 consensus sequence  ATCTGAAACATTGGAATTACCTACTATCAGTAGACCTACCAAACCAACCATATTGTCAGA
chimp 352_group 2 consensus sequence  ATCTGAAACATTGGAATTACCTACTATCAGTAGACCTACCAAACCAACCATATTGTCAGA
chimp 353_group 2 consensus sequence  ATCTGAAACATTGGAATTACCTACTATCAGTAGACCTACCAAACCAACCATATTGTCAGA
chimp 354_group 2 consensus sequence  ATCTGAAACATTGGAATTACCTACTATCAGTAGACCTACCAAACCAACCATATTGTCAGA

 chimp 350_group1 consensus sequence  GCCAAAGTTAGCATGGACAGACAAAGGTGGGGCAATCAAAACTGAAGTAAAGCAAACAAT
chimp 351_group 1 consensus sequence  GCCAAAGTTAGCATGGACAGACAAAGGTGGGGCAATCAAAACTGAAGTAAAGCAAACAAT
chimp 352_group 2 consensus sequence  GCCAAAGTTAGCATGGACAGACAAAGGTGGGGCAATCAAAACTGAAGTAAAGCAAACAAT
chimp 353_group 2 consensus sequence  GCCAAAGTTAGCATGGACAGACAAAGGTGGGGCAATCAAAACTGAAGTAAAGCAAACGAT
chimp 354_group 2 consensus sequence  GCCAAAGTTAGCATGGACAGACAAAGGTGGGGCAATCAAAACTGAAGTAAAGCAAACAAT

 chimp 350_group1 consensus sequence  CAAAATTATGGATCCTATTGAAGAAGAAGGGTCTACTGAGAAAAAGGTGCTGCCCTCTAG
chimp 351_group 1 consensus sequence  CAAAATTATGGATCCTATTGAAGAAGAAGAGTCTACTGAGAAAAAGGTGCTGCCCTCTAG
chimp 352_group 2 consensus sequence  CAAAATTATGGATCCTATTGAAGAAGAAGAGTCTACTGAGAAAAAGGTGCTGCCCTCTAG
chimp 353_group 2 consensus sequence  CAAAATTATGGATCCTATTGAAGAAGAAGAGTCTACTGAGAAAAAGGTGCTGCCCTCTAG
chimp 354_group 2 consensus sequence  CAAAATTATGGATCCTATTGAAGAAGAAGAGTCTACTGAGAAAAAGGTGCTGCCCTCTAG

 chimp 350_group1 consensus sequence  TGATGGGAAAACTCC-TGCAGAAAAGAAGTTGAAACCATCAACCAACACAAAAAAGAAAG
chimp 351_group 1 consensus sequence  TGATGGGAAAACTCC-TGCAGAAAAGAAGTTGAAACCATCAACCAACACAAAAAAGAAAG
chimp 352_group 2 consensus sequence  TGATGGGAAAACTCC-TGCAGAAAAGAAGTTGAAACCATCAACCAACACAAAAAAGAAAG
chimp 353_group 2 consensus sequence  TGATGGGAAAACTCCCTGCAGAAAAGAAGTTGAAACCATCAACCAACACAAAAAAGAAAG
chimp 354_group 2 consensus sequence  TGATGGGAAAACTCC-TGCAGAAAGGAAGTTGAAACCATCGACCAACACAAAAAAGAAAG

 chimp 350_group1 consensus sequence  TTTCATTTACACCGAATGAACCAGGAAAATATACAAAGTTGGAGAAAGATGCTCTAGACT
chimp 351_group 1 consensus sequence  TTTCATTTACACCGAATGAACCAGGAAAATATACAAAGTTGGAGAAAGATGCTCTAGACT
chimp 352_group 2 consensus sequence  TTTCATTTACACCGAATGAACCAGGAAAATATACAAAGTTGGAGAAAGATGCTCTAGACT
chimp 353_group 2 consensus sequence  TTTCATTTACACCGAATGAACCAGGAAAATATACAAAGTTGGAGAAAGATGCTCTAGACT
chimp 354_group 2 consensus sequence  TTTCATTTACACCGAATGAACCAGGAAAATATACAAAGTTGGAGAAAGATGCTCTAGACT

 chimp 350_group1 consensus sequence  TGCTTTCAGACAATGAAGAGGAAGATGCAGAATCCTCAATCTTAACCTTCGAAGAAAGAG
chimp 351_group 1 consensus sequence  TGCTTTCAGACAATGAAGAGGAAGATGCAGAATCCTCAATCTTAACCTTCGAAGAAAGAG
chimp 352_group 2 consensus sequence  TGCTTTCAGACAATGAAGAGGAAGATGCAGAATCCTCAATCTTAACCTTCGAAGAAAGAG
chimp 353_group 2 consensus sequence  TGCTTTCAGACAATGAAGAGGAAGATGCAGAATCCTCAATCTTAACCTTCGAAGAAAGAG
chimp 354_group 2 consensus sequence  TGCTTTCAGACAATGAAGAGGAAGATGCAGAATCCTCAATCTTAACCTTCGAAGAAAGAG

 chimp 350_group1 consensus sequence  ATACTTCATCATTAAGCATTGAAGCCAGACTAGAATCGATTGAGGAGAAATTAAGCATGA
chimp 351_group 1 consensus sequence  ATACTTCATCATTAAGCATTGAAGCCAGACTAGAATCGATTGAGGAGAAATTAAGCATGA
chimp 352_group 2 consensus sequence  ATACTTCATCATTAAGCATTGAAGCCAGACTAGAATCGATTGAGGAGAAATTAAGCATGA
chimp 353_group 2 consensus sequence  ATACTTCATCATTAAGCATTGAAGCCAGACTAGAATCGATTGAGGAGAAATTAAGCATGA
chimp 354_group 2 consensus sequence  ATACTTCATCATTAAGCATTGAAGCCAGACTAGAATCGATTGAGGAGAAATTAAGCATGA

 chimp 350_group1 consensus sequence  TACTAGGGCTATTAAGAACACTCAACATTGCTACAGCAGGACCCACAGCAGCAAGAGATG
chimp 351_group 1 consensus sequence  TACTAGGGCTATTAAGAACACTCAACATTGCTACAGCAGGACCCACAGCAGCAAGAGATG
chimp 352_group 2 consensus sequence  TACTAGGGCTATTAAGAACACTCAACATTGCTACAGCAGGACCCACAGCAGCAAGAGATG
chimp 353_group 2 consensus sequence  TACTAGGGCTATTAAGAACACTCAACATTGCTACAGCAGGACCCACAGCAGCAAGAGATG
chimp 354_group 2 consensus sequence  TACTAGGGCTATTAAGAACACTCAACATTGCTACAGCAGGACCCACAGCAGCAAGAGATG

 chimp 350_group1 consensus sequence  GGATCAGAGATGCAATGATTGGCATAAGGGAGGAACTAATAGCAGACATAATAAAAGAGG
chimp 351_group 1 consensus sequence  GGATCAGAGATGCAATGATTGGCATAAGGGAGGAACTAATAGCAGACATAATAAAAGAGG
chimp 352_group 2 consensus sequence  GGATCAGAGATGCAATGATTGGCATAAGGGAGGAACTAATAGCAGACATAATAAAAGAGG
chimp 353_group 2 consensus sequence  GGATCAGAGATGCAATGATTGGCATAAGGGAGGAACTAATAGCAGACATAATAAAAGAGG
chimp 354_group 2 consensus sequence  GGATCAGAGATGCAATGATTGGCATAAGGGAGGAACTAATAGCAGACATAATAAAAGAGG

 chimp 350_group1 consensus sequence  CCAAGGGAAAAGCAGCAGAAATGATGGAAGAGGGAATGAACCAAAGGACAAAAATAGGAA
chimp 351_group 1 consensus sequence  CCAAGGGAAAAGCAGCAGAAATGATGGAAGAGGAAATGAACCAAAGGACAAAAATAGGAA
chimp 352_group 2 consensus sequence  CCAAGGGAAAAGCAGCAGAAATGATGGAAGAGGAAATGAACCAAAGGACAAAAATAGGAA
chimp 353_group 2 consensus sequence  CCAAGGGAAAAGCAGCAGAAATGATGGAAGAGGAAATGAACCAAAGGACAAAAATAGGAA
chimp 354_group 2 consensus sequence  CCAAGGGAAAAGCAGCAGAAATGATGGAAGAGGAAATGAACCAAAGGACAAAAATAGGAA
```

```
 chimp 350_group1 consensus sequence   GCGGTAGTGTAAAATTAACTGAAAAGGCAAAGGAGCTCAACAAAATTGTTGAAGATGAAA
chimp 351_group 1 consensus sequence   ACGGTAGTGTAAAATTAACTGAAAAGGCAAAGGAGCTCAACAAAATTGTTGAAGATGAAA
chimp 352_group 2 consensus sequence   ACGGTAGTGTAAAATTAACTGAAAAGGCAAAGGAGCTCAACAAAATTGTTGAAGATGAAA
chimp 353_group 2 consensus sequence   ACGGTAGTGTAAAATTAACTGAAAAGGCAAAGGAGCTCAACAAAATTGTTGAAGATGAAA
chimp 354_group 2 consensus sequence   ACGGTAGTGTAAAATTAACTGAAAAGGCAAAGGAGCTCAACAAAATTGTTGAAGATGAAG

 chimp 350_group1 consensus sequence   GCACAAGTGGAGAATCTGAAGAAGAAGAAGAACTAAAAGACATACAGGAAAATAATCAAG
chimp 351_group 1 consensus sequence   GCACAAGTGGAGAATCTGAAGAAGAAGAAGAACTAAAAGACATGCAGGAAAATAATCAAG
chimp 352_group 2 consensus sequence   GCACAAGTGGAGAATCTGAAGAAGAAGAAGAACTAAAAGACATACAGGAAAATAATCAAG
chimp 353_group 2 consensus sequence   GCACAAGTGGAGAATCTGAAGAAGAAGAAGAACTAAAAGACATACAGGAAAATAATCAAG
chimp 354_group 2 consensus sequence   GCACAAGTGGAGAATCTGAAGAAGAAGAAGAACTAAAAGACATACAGGAAAATAATCAAG

 chimp 350_group1 consensus sequence   AAGATGACATTTACCAGTTAATTATGTAGTTTAATAAAAATAAACAATGGGACAAGTAAA
chimp 351_group 1 consensus sequence   AAGATGACATTTACCAGTTAATTATGTAGTTTAACAAAAATAAACAATGGGACAAGTAAA
chimp 352_group 2 consensus sequence   AAGATGACATTTACCAGTTAATTATGTAGTTTAATAAAAATAAACAATGGGACAAGTAAA
chimp 353_group 2 consensus sequence   AAGATGACATTTACCAGTTAATTATGTAGTTTAATAAAAATAAACAATGGGACAAGTAAA
chimp 354_group 2 consensus sequence   AAGATGACATTTACCAGTTAATTATGTAGTTTAATAAAAATAAACAATGGGACAAGTAAA

 chimp 350_group1 consensus sequence   A
chimp 351_group 1 consensus sequence   A
chimp 352_group 2 consensus sequence   A
chimp 353_group 2 consensus sequence   A
chimp 354_group 2 consensus sequence   A
```

Distance Matrix of P gene consensus sequences

	chimp 350_g...	chimp 351_g...	chimp 352_g...	chimp 353_g...	chimp 354_g...
chimp 350_group1 consens...		99.333%	99.666%	99.444%	99.333%
chimp 351_group 1 consen...	99.333%		99.666%	99.444%	99.333%
chimp 352_group 2 consen...	99.666%	99.666%		99.778%	99.666%
chimp 353_group 2 consen...	99.444%	99.444%	99.778%		99.444%
chimp 354_group 2 consen...	99.333%	99.333%	99.666%	99.444%	

References

Mackay, I. M., Bialasiewicz, S., Waliuzzaman, Z., Chidlow, G.R., Fegredo, D.C., Laingam, S., Adamson, P., Harnett, G.B., Rawlinson, W., Nissen, M.D. & Sloots, T.P. 2004. Use of the P Gene to Genotype Human Metapneumovirus Identifies 4 Viral Subtypes. *J Infect Dis*, 190, 1913-1918.

Reiche, J. & Schweiger, B. 2009. Genetic Variability of Group A Human Respiratory Syncytial Virus Strains Circulating in Germany from 1998 to 2007. *J Clin Microbiol*, 47, 1800-1810.

HMPV

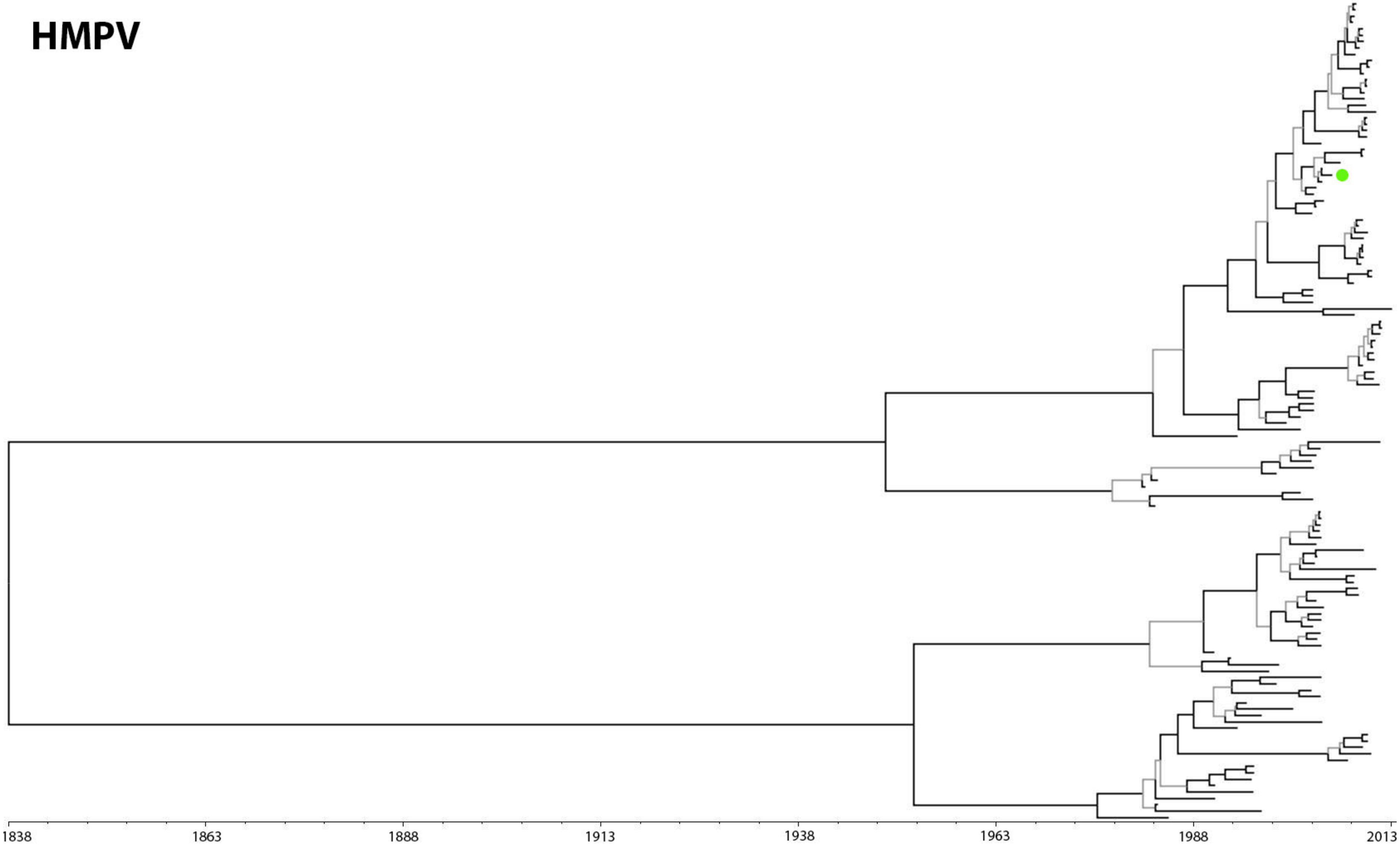

Supplementary Figure 1: Phylogenetic position of HMPV found in chimpanzees relative to human viruses sampled worldwide. The strain amplified from LWC outbreak in 2006 is indicated as a green colored dot. This tree is a maximum clade credibility tree generated from the output of Bayesian Markov chain Monte Carlo (BMCMC) analyses. Branch robustness was assessed through posterior probabilities. Branches with posterior probabilities > 0.95 are coloured in black.

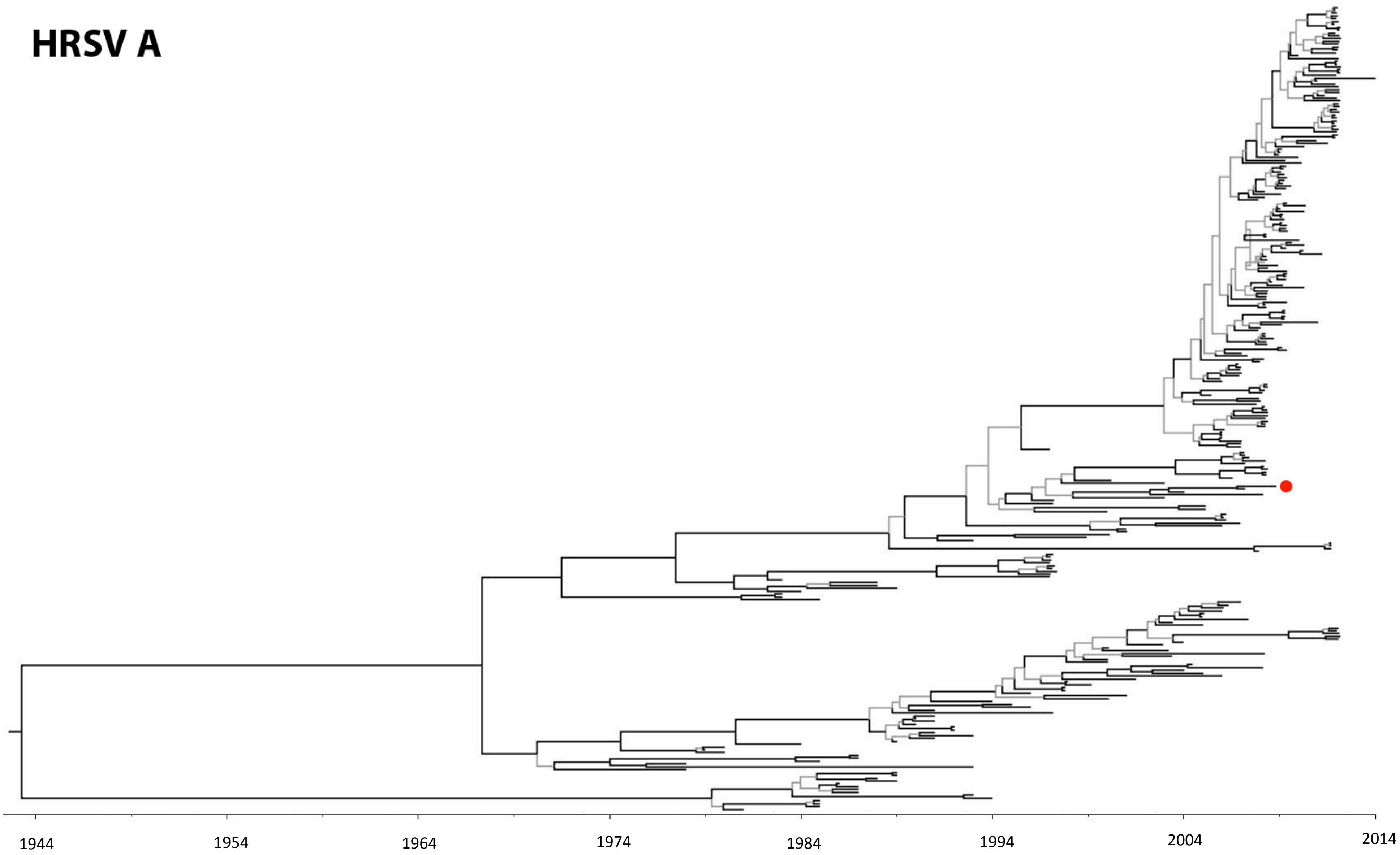

Supplementary Figure 2: Phylogenetic position of HRSV-A found in chimpanzees relative to human viruses sampled worldwide. The strain amplified from TNP outbreak in 2009 is indicated as a red colored dot. This tree is a maximum clade credibility tree generated from the output of Bayesian Markov chain Monte Carlo (BMCMC) analyses. Branch robustness was assessed through posterior probabilities. Branches with posterior probabilities > 0.95 are coloured in black.

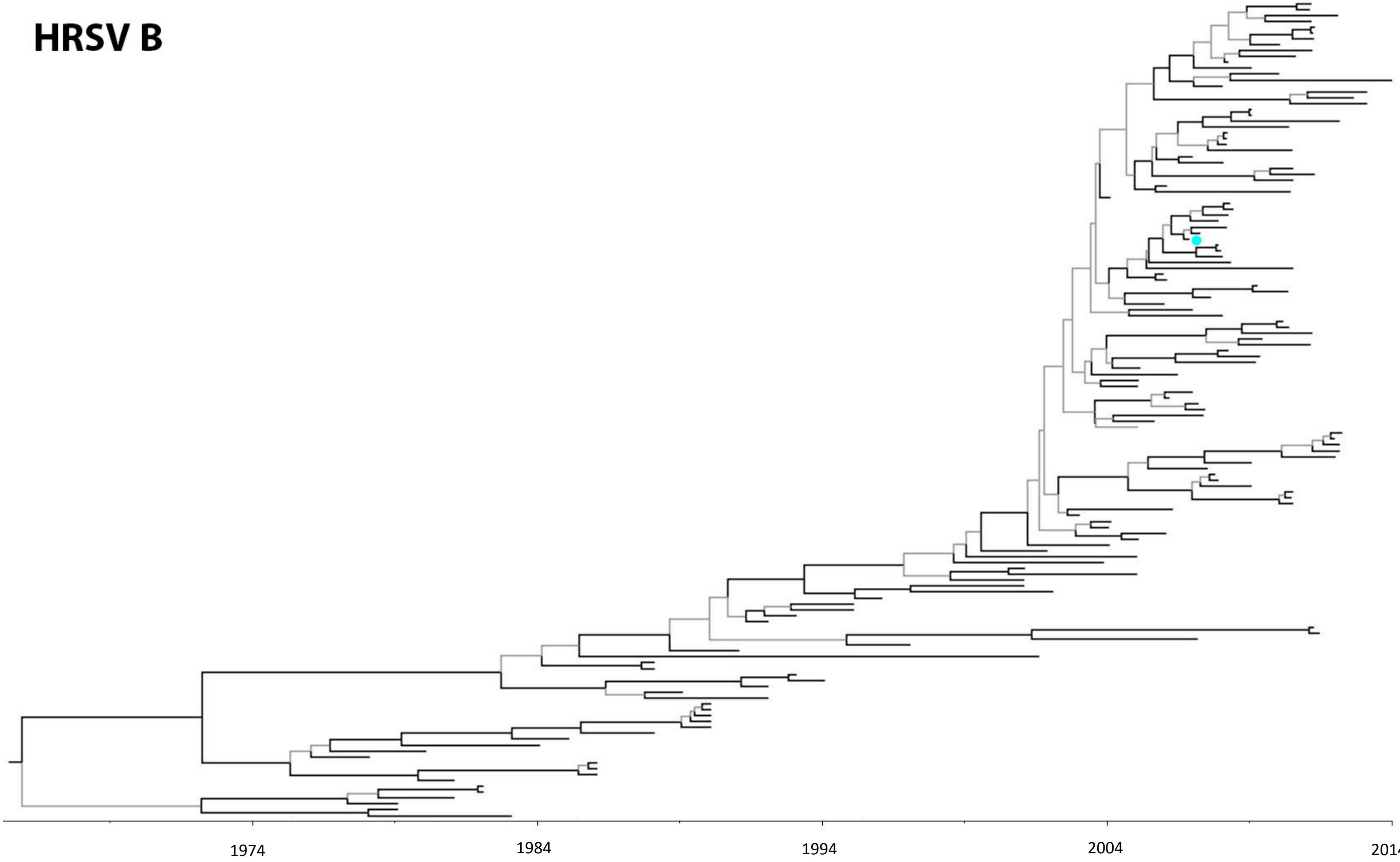

Supplementary Figure 3: Phylogenetic position of HRSV-B found in chimpanzees relative to human viruses sampled worldwide. The strain amplified from LWC outbreak in 2007 is indicated as a blue colored dot. This tree is a maximum clade credibility tree generated from the output of Bayesian Markov chain Monte Carlo (BMCMC) analyses. Branch robustness was assessed through posterior probabilities. Branches with posterior probabilities > 0.95 are colored in black.
